# Navigating discriminatory requests and refusals of healthcare workers: A Canadian-based inpatient hospital algorithm

**DOI:** 10.1177/09697330251374153

**Published:** 2025-09-06

**Authors:** Claudia Barned, Akosua Nwafor, Ann M. Heesters

**Affiliations:** 1 Department of Clinical and Organizational Ethics, 7989 University Health Network, Toronto, ON, Canada; 2 The Institute for Education Research (TIER), 7989 University Health Network, Toronto, ON, Canada; 3 Joint Center for Bioethics, Dalla Lana School of Public Health, University of Toronto, Toronto, ON, Canada; 4 Michener Institute of Education, 7989 University Health Network, Toronto, ON, Canada

**Keywords:** policy, organisational ethics, ethics and leadership/management, clinical ethics, discriminatory requests, healthcare algorithms

## Abstract

**Background:**

Healthcare workers are increasingly subject to violence, aggression, and discriminatory requests from patients and families, reflecting broader societal biases within healthcare settings. In response, some institutions have developed policies and decision-making tools to guide leaders in addressing these situations ethically, consistently, and in accordance with human rights obligations.

**Aim:**

This paper describes the revision of a previously published Caregiver Preference Algorithm to guide healthcare leaders in managing discriminatory patient requests. The goal was to create a more robust, accessible, and contextually sensitive tool to support decision-making.

**Research design:**

The algorithm was revised through a multi-phase quality improvement project aimed at enhancing support for both frontline clinicians and leadership.

**Participants and research context:**

The project was conducted at a large, multisite tertiary care hospital in Ontario, Canada. Interviews were completed with 27 healthcare workers from various clinical areas. Stakeholder consultations included clinical and operational leadership, legal counsel, patient relations, equity offices, patient partners, and frontline staff.

**Ethical considerations:**

This project was approved by the University Health Network’s Quality Improvement Review Committee [ID: QIRC 22-0378].

**Findings:**

The updated algorithm is structured around six key decision points: (1) patient acuity and capacity; (2) consideration of religious, cultural, or trauma-informed needs; (3) relevance of trainee or learner status; (4) whether the request violates the Human Rights Code; (5) the identity of the requester; and (6) the clinician’s willingness to continue care.

**Discussion:**

The revised algorithm integrates legal and ethical principles to help healthcare leaders navigate complex situations. It offers structured guidance while allowing flexibility to respond sensitively to diverse clinical contexts.

**Conclusion:**

This work contributes a practical, rights-based framework that can support healthcare institutions in ethically and consistently responding to discriminatory patient requests while protecting healthcare workers.

## Introduction

Healthcare workers are facing increasing levels of violence and aggression in the workplace, ranging from physical assault to verbal abuse and harassment.^1–5^ While this crisis has been widely acknowledged,^6–9^ less research and scholarly attention has been paid to the ways in which racism and other forms of discrimination are embedded within these encounters. Racialized healthcare workers, particularly those who are Black, or Indigenous are subjected not only to heightened aggression but also to racial slurs, stereotyping, and exclusion.^10–13^ In some cases, patients and their families go beyond verbal abuse, making discriminatory requests or refusals of care based on the healthcare worker’s race, ethnicity, or religious background. These situations surface profound ethical tensions and questions; for example, should healthcare institutions accommodate such requests when they appear to be grounded in strongly held patient preferences or values, or does doing so promote discrimination and infringe on the rights and well-being of healthcare workers?

Under the Ontario Human Rights Code (OHRC), healthcare workers have the right to work in an environment free from discrimination, harassment, and harm.^14^ Furthermore, healthcare institutions have legal and ethical obligations to ensure that their policies and practices do not expose employees to toxic work environments.^14,15^ When hospitals and healthcare organizations fail to address or, worse, condone discrimination from patients or their families—whether this takes the form of racial slurs, exclusionary treatment requests, or other forms of bias—they undermine their staff members’ dignity, safety, and well-being.^7^ In addition, they send a troubling message that patient preferences, including those that are rooted in bias, take precedence over the rights of healthcare workers.^7^ Historically, some hospitals have accommodated racist patient requests, allowing discrimination to persist, perhaps under a belief that doing so recognizes patients’ rights to self-determination.^16–18^ However, legal challenges have demonstrated that such actions are indefensible. In cases where staff members have sued their employers for failing to protect them from racial discrimination, courts have ruled in favor of the workers, affirming that institutions must actively prevent and address workplace discrimination.^19–21^ Protecting healthcare workers from racism, and other forms of discrimination, is not only a legal duty but an ethical obligation that healthcare managers and leaders cannot ignore given their responsibilities to provide safe working conditions for all who fall under their care and jurisdiction. Fulfilling this duty requires clear policies, an acknowledgement of institutional accountabilities, and a commitment to fostering workplaces that truly are inclusive and supportive.^7^

While patients have the right to make decisions about their own care, there are limits to the extent of their autonomy.^7^ These limits are defined by the need to maintain a safe, respectful, and professional environment for both staff members and other patients.^7^ In Ontario, Canada, healthcare workers navigating challenging workplace incidents often turn to hospital policies, their professional colleges or regulatory bodies, or relevant legislation—such as the OHRC^14^ and workplace safety laws—for guidance and support. Regulatory colleges, tasked with protecting the public interest, provide evidence-based practice standards to help professionals manage complex clinical issues, and the codes of conduct, or codes of ethics, applicable to members of self-regulating healthcare professions generally emphasize duties owed to patients, the public, and to other professionals. However, many have not explicitly acknowledged patient bias and discrimination as a practice issue that falls within their mandate. Consequently, healthcare workers are left without clear, profession-specific guidance on how to respond to discriminatory behavior. Instead, they must interpret and apply broader policies, such as those addressing the termination of therapeutic relationships due to violence or abuse, or guidelines on patient referrals. These resources, while helpful in some cases, do not adequately address the nuances of discrimination in clinical settings. In the absence of explicit direction from regulatory bodies, healthcare workers must rely on institutional policies or legal frameworks (e.g., OHRC) to navigate these difficult situations. This lack of standardized guidance creates inconsistencies in how discrimination is handled within and across health systems, and places the burden on individual workers, managers, and organizations to determine appropriate responses. Inconsistent responses within clinical contexts and across organizations can leave healthcare workers feeling unprotected, isolated, and devalued.^4,11,22–24^ The continued absence of institutional accountability may exacerbate feelings of anger, abandonment, and moral distress, especially among those with lived experiences of bias and exclusion.^6,18,25–27^

A growing body of research has examined this issue through the lens of organizational policy and guidelines, highlighting key recommendations for institutions seeking to navigate discriminatory requests arising in care provision.^28–34^ Anstey and Wright,^29^ for example, introduced a *Caregiver Preference Guideline* and accompanying decision making algorithm (*Caregiver Preference Algorithm*) at the University Health Network (UHN), a multi-site tertiary care academic hospital in Toronto, Canada. Their work offers a structured approach to managing patient requests for or refusals of care based on discriminatory preferences. The current paper builds on Anstey and Wright’s foundational work, outlining the changes in the local context that necessitated a revision of their algorithm and detailing the process taken to refine and implement the updated guideline. We present here the revised *Patient Bias and Preferences Algorithm*, which replaces Anstey and Wright’s *Caregiver Preference Algorithm* in our local context.

In this paper, we use the term *algorithm *to refer specifically to clinical decision support algorithms (CDS) as defined by Oxholm et al.^35^ CDS algorithms are intended to aid clinical problem-solving and improve both patient care and healthcare systems. Typically presented as flow diagrams or decision trees, CDS algorithms outline potential pathways for addressing a problem or guiding decision-making. Unlike logic-based automated algorithms rooted in the computational sciences, CDS algorithms are structured guides designed to support human judgment in complex, often context-sensitive situations, and are intended to help healthcare teams think through a problem systematically.

## The UHN context: The Caregiver Preference Guideline (2007) versus the Patient Bias and Preferences Guideline (2024)

The *Caregiver Preference Guideline* was developed to address patient requests for and refusals of specific healthcare workers at the University Health Network (UHN), a large research and teaching hospital network in Toronto, Ontario, Canada. The Bioethics Program, now the Department of Clinical and Organizational Ethics, in collaboration with what was then called the Office of Diversity and Mediation—now renamed the Inclusion, Diversity, Equity, Accessibility, and Anti-Racism (IDEAA) Office—created this tool to provide recommendations to managers on whether and how to respond to patient requests that could conflict with the institution’s anti-discrimination policies. It was prompted by numerous reported incidents, particularly cases of racism and other forms of discrimination, directed at staff members by patients and their family members or visitors. The tool was primarily created for use by managers to provide them with a starting point in addressing or navigating these scenarios, but it also provided them with a resource that delineates the multiple steps they need to take to ensure a thoughtful and safe response to distressing incidents. Clinical contexts in which these cases frequently emerged included the emergency department, the dialysis unit, specialized dementia unit, and geriatric care. For further information on how the *Caregiver Preference Algorithm* was designed and could be applied, see Anstey and Wright.^29^ The guideline was designed for UHN as it existed in 2007. Since then, UHN has expanded significantly, encompassing not only acute care but also rehabilitation and specialized cancer care spanning multiple sites. Additionally, the sociopolitical landscape has shifted dramatically, further highlighting the urgent need for a robust approach that is attentive to context, the complexity, and frequency of cases involving discriminatory patient requests, and the impact on staff well-being. In 2022, we launched a quality improvement (QI) project designed to ensure that the guideline would be comprehensive, responsive, and better equipped to support staff navigating complex ethical and discriminatory challenges in today’s healthcare environment.

Our quality improvement project used an evidence-informed approach to revising the *Caregiver Preference Guideline*. This included conducting: (1) a scoping review of the literature,^7^ (2) qualitative interviews with 27 healthcare workers from diverse clinical backgrounds across UHN, (3) a critical review of comparable health system policies, and (4) consultations with UHN representatives from various clinical departments, leadership groups, and support services.

### Interviews

Semi-structured interviews were conducted with 12 nurses (bedside and leadership), 5 occupational therapists, 2 social workers, 1 physiotherapist, 1 recreational therapist and 6 physicians (4 staff and 2 learners) from acute care, rehabilitation medicine, cancer care, and specialized clinics. Of the 27 interviews, 26 were virtual, and 1 was in-person.

### Policy review

The reviewed policies included: Mayo Clinic’s 5-Step Policy for Responding to Patient Bias,^36^ Penn State Health’s Patient Caregiver Preferences and Refusal of Care Based on Aspect of Diversity Policy,^37^ and Johns Hopkins’ Prohibiting Discrimination by Patients against Employees Policy.^38^

### Consultations

Consultations were conducted with representatives from the following departments and stakeholder groups across UHN: Legal Affairs, Labor Relations, Clinical Education, Medical Affairs, ED Physician Leadership, Internal Medicine Leadership, Clinical Directors from the ED & ICU, Psychiatric Nursing and Behavioral Specialists, Social Work Leadership and Practitioners, Patient Relations, Occupational Health & Safety Services, UHN Security Services, Patient Partners, the IDEAA office, and the Indigenous Health Program.

### Ethical Considerations

This project was reviewed and granted approval by UHN’s Quality Improvement Review Committee (QIRC) - ID#: 22-0378. Participants were provided with a written preamble outlining the project’s purpose, methods, and the voluntary nature of participation. They were assured that participation would not be connected to their employment status (i.e., their decision to participate or decline would remain confidential and would not be disclosed to supervisors, colleagues, or their employer). Information was provided regarding how data would be used, who would have access to it, and the measures in place to ensure confidentiality. All data were de-identified, coded, and stored securely, with access limited to the project team. Only the first author (CB) had access to identifiable participant information.

In 2024, UHN introduced the *Patient Bias & Preferences Guideline*, a revised framework designed to provide clearer guidance on responding to discriminatory requests and refusals for specific healthcare workers. The updated guideline includes information on the roles and responsibilities of key staff, a revised algorithm, and scripts to support staff in navigating challenging conversations. The revised *Patient Bias and Preferences Algorithm* builds on the foundational work in the *Caregiver Preferences Algorithm*, addresses previously identified gaps (Table 1) and incorporates critical considerations such as the needs of learners, the significance of patient cognition and mental status, and the relevance of the clinical contexts in which requests are made. These updates facilitate a more nuanced, comprehensive, approach that supports healthcare workers and recognizes institutional commitments to equity and non-discrimination. See Appendix A for the revised algorithm.

**Table 1.** Gaps in the C*aregiver Preference Algorithm* and their associated changes in the revised *Patient Bias & Preferences Algorithm*.

Gaps/identified opportunities in the *Caregiver Preference Algorithm*	Data Source	Finding/recommendation from source	Changes reflected in the *Patient Bias & Preferences Algorithm*
Provides high-level guidance for a general clinical context/ hospital setting	Interviews	Recommendation: • Changes need to address differences in response based on location and time (i.e., ED at night vs. on the weekend) (P20—Physician) • Need to implement differences in responses based on care setting (P23—Physician)	• Considers nuances that are applicable to diverse clinical contexts (including the emergency department) • Considers variations in clinical presentations/patients’ needs in relation to the request. Factors taken into account include emergent vs. urgent conditions, acute brain injuries and cognitive impairments, decision making capacity, and clinical factors influencing behaviour
Is agnostic about the source of the request. The algorithm therefore only acknowledges patients as the source of the request	Scoping review	Finding: • Healthcare workers experienced bias and discrimination from patients, and their families (7)	• Acknowledges that substitute decision makers, family members, visitors or essential care partners may also request or refuse particular clinicians on behalf of patients • Includes a pathway that considers a separate response for discriminatory requests/refusals from this group. The response includes nuances related to SDMs’ abilities to carry out their responsibilities while also preserving staff safety (e.g., through visitor restrictions)
	Policy review	Finding: • Mayo Clinic’s 5 step policy, John Hopkin’s policy for responding to bias incidents applies to both patients and accompanying persons (e.g., family, visitors)^36,38^	
	Interviews	Finding: • Clinicians have different obligations to patients and visitors in responding to bias (P27—Nursing leader)	
The targeted clinician is responsible for the initial investigation into the motivations for the change request	Interviews	Finding: • Targeted individual shouldn’t be the one to clarify the reasons/motivations for the change request (P23—Physician)	• Assigns the responsibility for investigation and response to the manager/supervisor • Includes prompts (available in the guideline) to assist managers in teasing out motivations/reasons for the request
Refers to healthcare workers as ‘caregivers’	Policy review	Finding: • Mayo Clinic & John Hopkins’ policies use the language of clinicians, and staff^36,38^ • Penn State’s policy refers to caregiving staff^37^	• Refers to ‘clinician’ and/or ‘healthcare worker’ to be inclusive of all staff members that might encounter these requests. This shift in language avoids ambiguity with respect to the way the word ‘caregiver’ is frequently used in everyday discourse (i.e. referring to a family member who provides care for their loved one or a paid helper who takes care of a child or a person with a disability or an illness)
Manager (accompanied by clinician) has an initial exploratory discussion with the patient to understand the reason behind the request	Interviews	Recommendation: • Targeted individual shouldn’t be the one to clarify the reasons/motivations for the change request (P23— Physician) • Managers should deal with and respond to patient bias—it is part of their scope/role (P15—Advance practice nurse) • Revised algorithm should encourage investigating the reasons for a biased request (P20—Physician)	• Discussion occurs between the manager and the requestor (patient/family member/visitor). The targeted clinician was omitted from this interaction as a form of harm reduction/staff safety measure • After the manager ascertains the nature of the request, the targeted clinician is invited to attend further discussions with the requestor if they are comfortable doing so
Assumes the manager is able to probe reasons for bias and identify discrimination or harassment	Interviews	Finding • Algorithm places significant decision-making power with managers/supervisors, relying heavily on their subjective judgement and skills which may vary per individual (P3—Physician)	• Acknowledges that these are difficult conversations to have and therefore includes prompts and clarifying questions to support managers in assessing biased statements and requests
	Scoping review	Finding: • Targeted staff often experience a lack of support from management when navigating conflict related to patient bias^7^ • Managers face educational barriers stemming from a lack of formal training / guidance on addressing patient bias and limited discussion on discrimination and racism within health professions education programs.^7^	
Does not immediately consider the potential trauma experienced by the clinician or the value of providing timely psychosocial support or referral to support services	Interviews	Recommendation: • Integrate discussions with the targeted clinician earlier in the algorithm (P21—Nursing leader) • Integrate trauma-informed and culturally sensitive principles using a top-down approach (P15—Advance practice nurse)	• Centers a trauma-informed approach and includes a check-in point with the targeted clinician early in the process
	Scoping review	Finding: • Patient bias impacts healthcare workers’ emotional and psychological wellbeing, self-perception, job satisfaction, and role performance^7^	
		Recommendation: • Offer resources and support for targeted clinicians: multi-disciplinary action committees, communication tools, training for self-defense, mental health supports, patient contracts, communication aids, support groups, sexual harassment offices (7)	
	Policy review	Finding: • Mayo Clinic’s policy incorporates empathic language and tips for responding to inappropriate requests, bias, and aggression^36^	
Does not include learners or consider the nuances of the learner experience	Interviews	Recommendation: • Student and learner specific considerations are necessary (P4—physiotherapist) • Outline process if patient continues to decline learner involvement (P4—Physiotherapist) • Preceptors need to be transparent about learner involvement prior to seeing patient. (P4—Physiotherapist) • Policy needs to clearly outline the different support needs and resources available to support learners (P23—Physician)	• Considers the specific needs of learners in a teaching hospital as well as the responsibilities of preceptors in ensuring that the learning environment is safe. It also considers the potential harm of continued involvement to the learner, while recognizing the importance of efforts to replace the missed learning opportunity
	Consultations	Recommendation: • Depending on the clinical area and the type of expertise/care needed, in some cases we may be required to advise patients that their care cannot be provided without learner involvement (Legal affairs, Medical affairs)	
	Scoping review	Recommendation: • Institutions should provide specialized training to prepare learners; institutions should also prioritize exemptions for leaners based on their needs in the moment (7)	
Does not provide guidance on how to respond if the request is accompanied by violence	Interviews	Recommendation: • Incorporate code whites as a possible avenue to address biased patient behaviour (P10—Nursing leader)	• Embeds current organizational protocols pertaining to violence and safety concerns. These include: (1) enlisting security, and calling a code white if appropriate, (2) relying on the workplace violence policy which outlines detailed steps for responding to violent incidents including invoking a behavioural safety alert (BSA), when warranted, and (3) filing an incident report • Describes additional supports that should be provided to learners should they experience violence. This includes those available through the hospital and their home institutions
	Policy review	Finding • Penn state outlines zero tolerance for threats, violence, disrespectful communication or harassment in their expectations of mutual respect. If a patient is threatening, staff are told to engage security staff or the manager/lead physician.^37^	
	Scoping review	Recommendation: • Inform security about any dangerous behaviour, physical attack or verbal abuse/threats (7) • Isolate the abusive patient/family member/visitor from other patients if/when necessary (7)	
Documentation recommendations are vague	Scoping review	Recommendation: • Create standardized methods of reporting and tracking bias and discrimination from patients (7)	• Specifies what should be documented (i.e., details of the incident), and where it should be documented (i.e., incident report, chart note, behaviour safety alert) • Specifies who has responsibility for documentation, and notes when it is the clinician’s responsibility as opposed to that of the manager
	Policy review	Finding: • Penn state established a centralized, transparent reporting process that includes the date of the event, the patient involved, a description of the event, and whether the request was granted and why^36^	
	Interviews	Recommendation: • Clarity on proper reporting mechanism (i.e., BSA vs. incident report) is needed (P16—Occupational therapist) • Dedicated reporting/documentation areas for incidents related to patient bias that are easily identifiable in charts would be helpful (P12—Social worker)	
The algorithm concludes with two major outcomes: 1) there is a change in clinician, or 2) the team figures out a way to work together given the circumstances Does not include the possibility that capable patients may/can choose to seek care elsewhere if their request for a different clinician is denied	Consultations	Finding: • Alternative discharge options may be available & appropriate based on the patient’s care needs/clinical risk assessment (General Internal Medicine (GIM) leadership & ED leadership)	• Three outcomes are identified: 1) there is a change in clinician (clinician wishes to reassign patient), 2) the targeted clinician decides to continue providing care and the patient is agreeable, or 3) the targeted clinician decides to continue providing care, but the patient still refuses • Acknowledges that there may be irreconcilable differences in values between the patient and the healthcare team. As a result, there is now a discharge option that includes the possibility of patients choosing to seek care elsewhere • Includes the option of a patient discharge without treatment following a risk assessment which determines whether the degree of risk (to the patient and to staff safety) is acceptable
		Recommendation: • Create an outcome where a clinical risk assessment is performed [i.e., risk of discharge without treatment is determined] (Legal affairs, GIM leadership)	
Does not specify which institutional supports/resources are available or should be made available to the clinicians affected by discriminatory requests or behaviour	Interviews	Recommendation: • Algorithm should acknowledge the support needs of individuals at different hierarchy levels (i.e., learners, staff, managers, directors) (P23—Physician)	• Includes additional consultative services that are available to support the clinician and/or wider team (for example, clinical and organizational ethics department; spiritual care; IDEAA office; employee and family assistance program)
		Finding: • Potential resources for clinicians include	
		• Ombudspeople, bioethics, social work (P5—Physician)	
		• WSIB, people and culture, bioethics, spiritual care (P21—Nursing leader)	
		• Crisis counsellors (P20—Physician)	
	Scoping review	Recommendation: • Offer resources and support for targeted clinicians: for example, multi-disciplinary action committees, communication tools, training for self-defense, mental health supports, patient contracts, communication aids, support groups, sexual harassment offices (7) • Provide debriefs, expectation setting, role clarity support^7^	
If the patient maintains their request for a change, the manager assesses the operational feasibility of the discriminatory change request prior to asking the clinician if they wish to continue caring for the patient or be reassigned to another patient. The assessment of operational feasibility therefore does not account for the clinician’s potential trauma or psychological/emotional state or needs	Interviews	Recommendation: • Algorithm to consider more than just operational feasibility (i.e., the clinician’s experience/level of trauma/impact to the therapeutic relationship) (P19—Occupational therapist) • There is an overemphasis on considerations of operational feasibility. Should make transfers of care to protect staff members (P19—Occupational therapist) • Remove second consideration of operational feasibility as it implies that some bigoted requests might be accommodated if patient maintains biased request (P27—Nursing leader)	• After informing the patient of their inappropriate request and the discriminatory nature behind it, the revised algorithm prompts the manager to immediately check in with the clinician to assess for trauma and to ascertain the needs or well-being of the clinician • Includes a transfer of care component rooted in a trauma-informed approach. This change notes that if the clinician is traumatized or negatively affected by the interaction, the manager ought to find a new clinician to take over the patient’s care, as soon as possible/feasible
If the targeted clinician wishes to excuse themselves, the manager meets with the patient and communicates that there will be a change in clinician	Interviews	Recommendation: • Inform the patient that the change in caregiver was due to the clinician’s request/protection of staff safety as opposed to their request/refusal. (P27—Nursing leader)	• Includes specific language about what to communicate to the patient/family after the clinician has decided to reassign the patient/be recused. More specifically, the revision notes that the manager is to inform the patient that the change in clinician is not on account of their request but rather to protect staff from inappropriate discriminatory behaviour
	Scoping review	Recommendation: • Supervisors/managers should reassign and/or transfer harmful patients and explain the transfer of care (7)	
Communication about the incident happens at the group level – the reason for clinician reassignment is shared with unit staff	Policy review	Recommendation • Penn State’s policy notes that the decision to accommodate or not should be made by members of the healthcare team most familiar with the patients’ cultural, religious, and social background^36^	• Includes communication at the group level and at the individual level. In addition to communicating the reason for change with unit staff, the revision notes that the new clinician taking over the patient’s care should be informed privately about the transfer of care and any nuances related to the patient’s behaviour • An additional check-in point with the targeted clinician is added to the procedure during which the manager shares additional institutional supports that are available, or could be helpful (e.g., Spiritual care, clinical and organizational ethics, inclusion, diversity, equity, accessibility & anti-racism (IDEAA), employee assistance program (EAP), information on how to file a WSIB claim) • Offers the option of having a larger team debrief with the rest of the unit (if the affected clinician desires)
	Scoping review	Recommendation: • Management should reassign and/or transfer harmful patients and explain the reason for the transfer of care (7) • Teams should debrief as a unit after incidents of patient bias (7) • Teams should collaborate to create a plan to protect targeted individuals (7)	
	Interviews	Recommendation: • Before assigning a new clinician to the patient, inform the potential clinician about the patient’s behaviour/request and provide the option to either accept or reject the assignment (P9—Nurse)	
The sole legislative framework underpinning the algorithm is the OHRC.	Interviews	Recommendation: • The organization should make the institution’s values on diversity, equity and inclusion, anti-racism and anti-oppression, visible and prominent (P12—Social worker) • The organization should embed a zero tolerance approach towards racism and discrimination (P20 - Physician)	• Relies on both the OHRC and the OHSA to outline the institution’s responsibilities/obligations to protect healthcare workers and learners • Includes references to institutional policies that are rooted in legislative frameworks (e.g., Workplace violence policy)
	Consultations	Recommendation: • Ensure features of the OHSA, and UHN’s refusal to work policy are balanced with the employee’s rights under the OHRC (i.e., noting that under certain situations it might be unsafe for the patient should the clinician refuse to work. In these instances, the manager must find ways to support the clinician until a safe transfer of care is possible (Legal affairs, IDEAA, Occupational health & safety, Labor relations)	

## Applying the Patient Bias and Preferences Algorithm

The *Patient Bias & Preferences Algorithm* is a five-page decision-making tool with six key decision points, designed to guide responses when a patient’s request or refusal of a specific healthcare worker is tied to consent to treatment. The process begins with the affected clinician informing their manager of the request, thereby initiating a discussion to clarify the requestor’s values, beliefs, and reasoning. To minimize harm or retraumatization of the affected healthcare worker, the manager—rather than the clinician—leads further discussions with the requestor. This ensures that explicit concerns about care can be addressed while also offering the requestor an opportunity to reconsider and withdraw their request if it is discriminatory in nature. In cases where requests or refusals involve slurs, epithets, or violent behaviour, the discussion focuses less on exploring rationale and more on reinforcing the institution’s anti-discrimination policies and expectations for respectful conduct.

At this stage, the manager, with input from the clinician, reaches the first decision point, assessing the requestor’s preference in light of their clinical presentation:

### Acuity and capacity: Does the patient have urgent or emergent medical needs? Are they capable of refusing treatment or imposing conditions on their care?

Assessing a patient’s acuity is critical to determining whether immediate health-related needs take precedence. While healthcare workers are not expected to tolerate discriminatory behaviour, they also have ethical duties to provide care, particularly in emergencies. In crisis situations, the obligation to deliver treatment can override the need for discussions about the request, with priority being placed on stabilizing the patient.

The updated algorithm explicitly ties the refusal of a specific healthcare worker—or the request for a particular provider—to consent to treatment. This requires assessing the patient’s capacity to make treatment decisions and considering whether they understand the implications of their request or refusal. Importantly, capacity is assessed in relation to a specific treatment decision and is not treated as a global determination. This decision point also considers how a patient’s stability and capacity intersect, evaluating whether clinical factors (e.g., cognitive impairment, delirium, or mental health crisis) are influencing their ability to appreciate both the clinical and psychosocial consequences of their preferences—including potential harm to themselves or staff.

### Religious, cultural, and trauma-related needs: Are there reasonable, non-discriminatory reasons for the request/refusal?

The second key decision point involves an exploration of reasonable, non-discriminatory, concerns that may affect the patient’s care experience directly. If the requestor has not yet articulated specific care-related needs, this step provides an opportunity to clarify whether their request is linked to accommodations protected under human rights legislation such as the Ontario Human Rights Code. These may include cultural, spiritual, or religious considerations, as well as past traumatic experiences, that could influence their degree of comfort with certain providers. The goal is to determine whether the request aligns with trauma-informed or culturally sensitive care, which can enhance patient safety, trust, and overall well-being. However, it is also recognized that some religious, cultural, or trauma-related requests may still be shaped by implicit bias or discriminatory beliefs, meaning that not all such requests automatically warrant accommodation.

If a request is determined to be rooted in a traumatic experience or religious-based need, the manager can communicate this to the affected healthcare worker, clarifying that the decision is not based on discriminatory grounds but rather on a request that supports the patient’s care journey in a meaningful way. Likewise, the patient is to be informed that the request is being accommodated due to legitimate clinical or psychosocial factors rather than personal preferences alone.

### Learner status—is the refusal based on the clinician’s “trainee” status?

As a teaching hospital, UHN has a responsibility to protect and support the education of its diverse cohort of health professions learners. Learners may experience refusals of care based solely on their trainee status, aspects of their identity, or both. This decision point ascertains whether the refusal is primarily due to the learner’s status as a learner and requires a check-in with the learner to evaluate their level of harm, comfort, and willingness to proceed. The patient’s interest in directing their own care must be weighed against (1) the institution’s obligation to provide teaching and learning opportunities to those in training and (2) its duty to ensure that future patients have a well-prepared workforce available to meet their needs. While capable patients may exercise their rights to refuse care, or care from specific healthcare providers, teaching hospitals must establish clear boundaries regarding the treatment of learners, ensuring that necessary support systems are in place to foster their success and well-being. Although nuances exist across various contexts, disciplines, and professions, in cases where a patient refuses care from a learner, it is still the capable patient’s right to wait for a fully licensed provider, even if this results in delayed treatment. Institutions should take steps to determine whether these refusals are rooted in discriminatory biases and ensure that learners are protected from harm and exclusion while training in a clinical environment.

### Discrimination or harassment—does the request/refusal discriminate against or harass the clinician?

This decision point determines whether the patient’s request or refusal is rooted in bias or discrimination under any of the 14 protected grounds of the OHRC (which references race, ancestry, place of origin, color, ethnic origin, citizenship, creed, sex, sexual orientation, age, record of offenses, marital status, family status, and disability). The OHRC legally protects individuals from discrimination based on these grounds, which determine the institution’s legal and ethical obligations in responding to such requests. Appendix B of the *Patient Bias and Preferences Guideline* includes prompts and guiding questions to help managers and decision-makers tease out the particulars of the request or refusal. The goal is to differentiate between reasonable, patient-centered, concerns (e.g., trauma-informed needs, religious accommodations) and discriminatory requests that violate institutional policies and obligations.

### Source of the request—who is the requestor and how do our obligations change in reference to the source of the request?

The next decision point clarifies who is making the request or refusal—the patient, a family member, or another party—as this helps determine the legal and ethical obligations of the institution. While healthcare workers have clear duties of care with respect to their patients, this obligation does not extend in the same way to family members or visitors. However, complexities arise when a family member serves as a substitute decision-maker (SDM) or when they hold Power of Attorney for Personal Care, as they then are legally authorized decision-makers (LARs) for incapable patients. In these cases, the institution must both uphold and respect their decision-making rights, even if their behaviors or attitudes are problematic. This requires careful navigation; respect for the SDM’s role must be balanced against the institutional and leadership responsibility to ensure that discriminatory behavior does not go unaddressed. If the request or refusal comes from a non-decision-making family member or visitor, the institution has greater flexibility to take corrective action, including setting firm boundaries on unacceptable conduct. Depending on the situation, responses may range from education and dialogue to removing privileges for disruptive visitors if their behaviour creates a toxic or unsafe environment for healthcare staff members.

### Agency of the clinician—does the clinician wish to excuse themselves/reassign patient or to continue providing care?

An important goal of the algorithm is to ensure that affected healthcare workers have agency in deciding how they wish to proceed. At this decision point, the clinician is given the choice to:

(a) Continue providing care, or
(b) Request reassignment (if operationally feasible)

Irrespective of their choice, the manager must express solidarity and support for the clinician. If the clinician opts to continue care, the manager should reinforce the institution’s commitment to a safe and discrimination-free workplace and affirm the clinician’s competence and professionalism to both the staff member and the requestor. This ensures that the decision is not seen as validating the discriminatory request, but rather as the result of clinician’s autonomous choice to continue to support the patient. If the clinician requests reassignment, the institution should facilitate the transition without validating the bias underlying the request. The manager must explicitly communicate to the requestor that the change is not a concession to their discriminatory demand, but rather a protective measure to safeguard the well-being of the staff member. Additionally, the affected clinician should be provided with access to institutional supports to help process the emotional and psychological impact of the incident. This may include referral to an Employee Assistance Program or other resources. By emphasizing a commitment to both agency and support, this approach ensures that healthcare workers are empowered rather than burdened by these challenging encounters, and it reinforces the organization’s commitment to equity, safety, and professional dignity.

## Discussion

This paper describes the revisions made to Anstey and Wright’s^29^ algorithm and the processes available to respond to discriminatory requests or refusals of care in a Canadian tertiary care academic hospital. In this paper, we note important contextual factors that are relevant to the assessment of whether, how, and when one should respond to, or accommodate, a request or refusal that is discriminatory in nature. The algorithm was designed specifically for adult inpatient care environments. Establishing institutional practices and recommendations for responding to patient bias in other care contexts (e.g., in pediatric or outpatient settings) requires consideration of population-specific factors and clinical nuances. Notably, what UHN has instituted, and what has historically existed, is a guideline not a policy. A guideline was chosen during the revision due to considerations about what is enforceable, and what is recommended. Organizational accountability and responsibility differ depending on whether a policy or a guideline is adopted. For example, if an institution were to establish a policy, there would be an obligation to inform patients and families about the existence of that policy, as well as the parameters under which a decision to accommodate or not to accommodate was made. Some clarity would be gained, but significant context-specific flexibility and adaptability would be lost.

The *Patient Bias and Preferences Algorithm* incorporates recommendations from the literature regarding the importance of the agency of the clinician, especially learners,^39–44^ the need for learner-specific guidance and resources,^28,40,42,45^ clear documentation processes,^29,32,43,46–48^ and better communication with respect to the existence of these guidelines with patients.^29,43,48,49^ In addition, our quality improvement project identified the following 7 needs: (1) specific documentation based on the type of request or refusal made (i.e., incident reports, clinician notes in the electronic medical record); (2) data collection on the various types of requests/refusals and resolution, frequency of occurrence, identity of the requestor, clinical setting in question and impact on the clinician; (3) clear escalation pathways (including when to escalate and to whom); (4) identification of key organizational supports (such as Clinical and Organizational Ethics, Spiritual Care, Administrators on Site, Division Heads); (5) identification of the role of the preceptor and learner-specific resources; (6) context specific scripts and speaking prompts to support conversations with patients and family members at different points in the process; and (7) information on related organizational policies and processes (e.g., Workplace Violence and Harassment, Refusal to Work).

Whereas Anstey and Wright’s^29^ foundational work is grounded primarily in human rights legislation, the revised guideline and algorithm incorporate both human rights legislation and occupational health and safety legislation as fundamental legal considerations with implications for the quality of staff experiences, employee well-being, and workforce retention. New federal legislation provides additional legal grounding to protect healthcare workers from violence. Bill C-3, which received royal assent in December 2021 created a new intimidation offence for behaviour intended to provoke fear in a healthcare professional in order to impede the performance of their duties.^50^ Although not included in the revised guideline or algorithm, this new legislation includes legal ramifications for discriminatory behaviour from patients and families, beyond visitor restrictions.

The revised algorithm serves both informational and instructional purposes, enabling point-of-care healthcare workers to easily identify available procedural options and relevant resources. It is also intended to support managers and healthcare leaders in guiding their responses in ways that are sensitive to the needs and capacities of their patients and staff members. While the academic literature on this topic is expanding, there remains a significant need for increased knowledge dissemination and skill building in this area, particularly as interpersonal tensions continue to escalate.

Importantly, we do not intend the algorithm to be directly transposed to different healthcare contexts, but rather to prompt reflection, recognizing the shared challenges while respecting local differences. For settings outside of Ontario, we recommend that institutions draw on their own occupational health and safety legislation, anti-discrimination laws, and relevant human rights frameworks (which in many Canadian provinces, and in other countries reflect the protections outlined in Ontario’s policies), to develop equivalent approaches tailored to their specific regulatory and institutional contexts. Additionally, other hospitals often have comparable functions, even if named differently, such as committees to address violent behaviors or offices addressing health and safety, as well as anti-discrimination. Furthermore, many codes are standardized across institutions (e.g., Code Blue).

## Conclusion

Healthcare workers, and the patients they serve, come from diverse backgrounds and it is impossible for healthcare institutions to be insulated entirely from the disturbing expressions of bias and interpersonal violence that occur in the wider community. This has always been true; but what has changed in recent years, perhaps in recognition of increasing incidents of aggression toward healthcare workers and the increased complexity of patient circumstances, is the expectation that healthcare leaders pay greater attention to the wellbeing of the workforce. In addition, it is now understood that employee recruitment and retention is linked inextricably to efforts to attend to staff members’ well-being, and that efforts to reduce non-physical harms are essential components of sustainable and ethically attuned institutions. Patient declarations of values, professional and institutional codes of ethics, and codes of conduct, are worthy, but insufficient, tools for advancing what is now described as the quadruple aim of healthcare.^51,52^ Put another way, an ethical workplace is one which strives for safety for all, and that entails a commitment to psychological as well as physical safety and respect for cultural diversity.

Our hope is that UHN’s revised and expanded *Patient Bias and Preferences Guideline* will assist those who struggle to find a practical, intuitive, and inclusive way of balancing patient and family requests with the responsibility to ensure a safe and respectful environment in which all are free to do their best work and where patients receive the highest quality of care.

## Supplemental Material

**Supplemental Material**—Navigating discriminatory requests and refusals of healthcare workers: A Canadian-based inpatient hospital algorithm Supplemental Material for Navigating discriminatory requests and refusals of healthcare workers: A Canadian-based inpatient hospital algorithm by Claudia Barned, Akosua Nwafor, and Ann Heesters in Nursing Ethics.

**Supplemental Material**—Navigating discriminatory requests and refusals of healthcare workers: A Canadian-based inpatient hospital algorithm Supplemental Material for Navigating discriminatory requests and refusals of healthcare workers: A Canadian-based inpatient hospital algorithm by Claudia Barned, Akosua Nwafor, and Ann Heesters in Nursing Ethics.
