# Supplementary material for: Navigating discriminatory requests and refusals of healthcare workers: A Canadian-based inpatient hospital algorithm
Source: Nurs Ethics. 2025 Sep 6;33(2):344–64. doi: 10.1177/09697330251374153 (PMC13009228; doi:10.1177/09697330251374153)
Supplement: Supplemental Material—Navigating discriminatory requests and refusals of healthcare workers: A Canadian-based inpatient hospital algorithm [file sj-pdf-1-nej-10.1177_09697330251374153.pdf]

Navigating Requests for Specific Healthcare Workers

Patient / family member / ECP requests a change in clinician and withholds consent to treatment if request is not granted

↓

Clinician or colleague informs supervisor / manager of request / preference

↓

Supervisor / manager / charge nurse / AOS clarifies reason for change and inquires if there are other concerns that might need addressing
(See Appendix A in Guideline for prompts)

↓

Is the patient stable and capable?

NO ↓ ***Consider the needs of the patient and focus on achieving stability***

YES → ***Proceed to next page if the patient is stable and capable*** →

A) Does the patient have acute clinical factors affecting their behaviour?

Assess if there are ways to optimize / treat reversible clinical disorders (ex. delirium, psychosis). Consult CL Psych, Substance Use consult team (SUITs), Geriatric Medicine, Peer Support Workers as appropriate. →

For patients with acute brain injuries, cognitive impairment, treatment resistant mental health conditions or any other conditions that cannot be optimized, **negotiate with the patient** to establish mutually acceptable conditions for providing care. If the patient lacks decision-making capacity, involve the SDM in discussions.

See Question C below

B) Does the patient have urgent/emergent health needs?

Treat clinically/accommodate. Patient should still be informed that UHN condemns hateful conduct and any such behaviour contravenes UHN's anti-discrimination policy.

If patient is not urgent and has no emergent health needs, assess if ED level of care is warranted. If not, assess whether discharge is appropriate. →

Options for responding include:

- Establish mutually acceptable expectations and conditions for providing patient care
- If other clinicians are available, and it is reasonable/ feasible to switch, reassign patient.
- If only one clinician is available, or if the clinician does not wish to reallocate patients, negotiate with the patient to allow for care to be provided until another clinician is able to take over.

C) Does the patient lack decision making capacity?

Involve the SDM, discuss UHN's anti-discrimination policy with both the patient and SDM, seek consent of the SDM to proceed with assigned clinician (should the clinician wish to continue providing care) and boundary set with the patient.

In the absence of SDMs, contact the PG&T to obtain consent. If the PG&T is unavailable, and emergency care is required, proceed on an emergency basis without consent as noted in section 25 (1-4) of the HCCA, and UHN's Consent to Treatment Policy. →

In an emergency context: If SDM refuses treatment by a specific provider due to discrimination under any of the 14 protected grounds, and is not making decisions in accordance with the patient's wishes or in their best interests, one can proceed on an emergency basis without consent.

Non-emergency context: Ensure that the SDM understands their responsibilities under the HCCA. Consult Bioethics and Legal. Consider submitting a Form G to the Consent & Capacity Board.

**Proceed to Page 6 if request is from family/ECP*

Your patient is stable and capable

Is the request based on religious, cultural, spiritual or trauma concerns / needs?

YES

Make best efforts to accommodate the request based on the religious, cultural, spiritual or trauma considerations shared.

If staffing concerns do not permit an immediate accommodation, explain the options for clinicians and involve the patient in shared decision making.

↓

If the patient chooses to wait until their request can be accommodated, manager and/or physician lead must:

1. Inform patient of the implications of waiting, and
2. Relay potential consequences due to the delay in receiving care (i.e., urgency of treatment should be weighed in relation to risks of delay).

Manager or clinician to document request, rationale and decision in patient's chart

NO

Is the request based on one's status as a learner?

YES — **Supervisor/Preceptor** to:

1. Determine risk of involvement/assess level of potential harm to learner
2. Check in with the learner to gauge interest in proceeding.

→ **Supervisor/Preceptor** to communicate the following to the patient/family:

- UHN is a teaching hospital and learners are involved in all aspects of care. Depending on the clinical context, care might not be possible without learner involvement.
- Clarify learner role/scope and level of supervision at UHN
- Note the capabilities and qualifications of the learner
- Ask about any perceived discomfort regarding the learner's presence

Once this has been discussed with the patient, ask for consent for learner to participate in the patient's care under these conditions.

↓

If the patient does not consent to learner involvement, the supervisor should take over care and make reasonable efforts to replace the missed learning opportunity.

If a patient refuses to have a learner involved and chooses to wait for the supervisor/preceptor, the patient must be informed of the implications and potential consequences of the delay in receiving care.

↓

Supervisor/preceptor to document request, rationale and decision in patient chart

NO ↓

Is the request rooted in bias or discrimination based on any of the 14 protected grounds under the Ontario Human Rights Code? (i.e., race, ancestry, place or origin, color, ethnic origin, citizenship, creed, sex, sexual orientation, age, record of offenses, marital status, family status, or disability)

YES → ***If yes, proceed to next page*** →

NO

If the request is based on a preference that will not impact the patient's health or care trajectory, assess whether fulfilling the request is operationally feasible (e.g., a request from a female patient seeking a female urologist if possible, due to comfort).

→

- If there are staffing constraints, tell the patient why the request cannot be fulfilled at this time and explain the current options for care.
- If no staffing constraints, and a switch could be reasonably provided without negatively affecting the care of other patients, switch.

→ **Manager or clinician to document request, rationale, and decision in patient chart**

Is the request rooted in bias or discrimination based on any of the 14 protected grounds under the Ontario Human Rights Code (See list on page 4)?
(See Appendix B in Guideline for prompts to guide conversation)

YES

If requestor is a patient

Manager/supervisor (with necessary supports) has boundary-setting conversation with the patient, in the presence of the affected clinician (if clinician desires).

Manager/supervisor informs patient that the request is inappropriate given its discriminatory nature and shares that:

1. This type of request asks UHN to participate/collude in discriminatory behaviour prohibited by Ontario's Human Rights Code.
2. UHN policy prohibits discrimination, harassment and violence against staff.
3. All UHN staff members are fully capable and qualified to deliver excellent care.

→

In a private space, check-in with affected clinician to assess level of trauma and provide support.

After assessing the clinician's wellbeing, the manager should reaffirm commitment to UHN policies and the Ontario Human Rights Code, and let the clinician know that the request was inappropriate and unacceptable.

→

Manager/supervisor should support the clinician in filing an incident report.

- **If request is accompanied by violence** → **Call Security and if appropriate, call a code White.** Follow steps outlined in UHN's workplace violence policy, and if situation meets requirement for BSA, follow steps in respected policy.
- **If affected clinician is a learner, trigger all learner resources and supports within UHN and home institution/school**
- **Support the agency of the affected clinician by giving them the option to continue caring for the patient or reassign.**

If request comes from family / visitor / ECP

Manager/supervisor informs family/visitor/ECP that the request is inappropriate given its discriminatory nature.

Manager/supervisor shares that:

1. UHN will not participate/collude in discriminatory behaviour prohibited by Ontario's Human Rights Code.
2. UHN policy prohibits discrimination and harassment against staff.
3. All UHN staff members are fully capable and qualified to deliver excellent care.
4. UHN has an obligation to provide a safe workplace for its staff. At the discretion of the manager, persistent discriminatory behaviour may result in visitation restrictions. (Ensure verbal conversation with documentation to follow).

→

Manager/supervisor to consider if any UHN support services would benefit family/ECP (e.g. Patient Relations, IHP, Bioethics).

- **If the discriminatory behaviour escalates, and is accompanied by violence, call security for support and/or to escort the family member/visitor off the premises. Document in the patient's chart and complete any necessary reporting as per the steps in the workplace violence policy.** → If the family member or visitor engaging in discriminatory behaviour is also the SDM of the patient, ensure that any restrictions imposed does not impede their ability to perform their role.
- If visitor restrictions have been enforced, the manager should escalate to the clinical director. Follow steps outlined in visitor policy and collaborate with security re unit based protocols. → Ensure any restrictions on visitation have been communicated verbally and in writing, noting the reasons for restrictions, that they are not absolute and can be appealed.

For next steps for clinician to take, proceed to next page →

The clinician can decide to A) Continue providing care OR B) Reassign patient

B) ***Reassign patient, proceed to next page*** →

A) Continue providing care

Manager/supervisor (supported by Clinical Director, Division Head and/or Bioethics, if necessary) to inform patient that:

1. Their request will not be accommodated
2. All UHN staff are capable and qualified to deliver excellent care
3. All staff have a right to work in an environment that is free of discrimination, harassment, and violence.
4. The patient bill of rights & responsibilities states that there are expectations for how patients conduct themselves while at UHN (manager could provide patient with UHN's Patient Bill of Rights and Responsibilities).

If patient agrees to receive care from the clinician

Manager/supervisor to document in patient's chart

Provide ongoing staff support by debriefing with affected clinician (invite Bioethics, IDEAA, IHP, and Spiritual Care, if desired).

If the patient still refuses to be cared for by the clinician or withdraws their consent to the proposed treatment

Manager/supervisor and/or MRP to evaluate risk to staff and patient of continued hospitalization.

Manager/supervisor to inform the patient that a switch will not be provided. Discuss the risks/ benefits and implications of continued refusal. Inform the patient that their options are:

1. Accept care by our clinician

2. Seek care elsewhere.

Document in the patient's chart. If the patient chooses to seek care elsewhere, a referral should be offered as part of their discharge. Inform senior management and MRP of the above.

If patient continues to refuse care from the clinician, clinical team should conduct risk assessment and weigh the following options:

A) Risk of discharge without treatment found to be low/acceptable.

MRP informs patient that their clinical needs can be addressed elsewhere and discusses risks, benefits and implications of leaving without treatment. MRP documents discussion and the patient's response.

MRP prepares discharge orders (+referral) and informs the patient of their options to seek care else. If the patient refuses to leave, call security for assistance in escorting the patient off the premises.

Document the interaction in the patient's chart.

B) Where there is some degree of risk

MRP to weigh the appropriateness of a discharge at risk given factors relevant to the patient (behaviours, risk to staff, risk to patient, specialist expertise offered at treating site).

Where the risk to staff is severe, and the risk to patient of continued hospitalization outweighs the benefits, a discharge at risk may occur.

Where the risk to staff is severe, and the risk to patient is high, assess feasibility / appropriateness of a transfer to another receiving unit or organization.

If conducting a discharge at risk or transfer, collaborate with Clinical Director, Legal, and Department Head.

MRP To document in patient's chart

Where the care required by the patient is life or limb, OR is not available elsewhere.

MRP collaborates with clinical director, department head and legal to weigh if there are nuances to the patient's context that outweigh their behaviours/risk to staff.

If the patient requires specialized care only accessible at the treating site, or if the patient's social history prevents an accessible discharge, find an alternative UHN provider, however, ensure that the patient is made aware that their behaviour and request are unacceptable.

The clinician can decide to:

B) Reassign Patient

Manager/supervisor initiates transfer of care or implements safety measures to support the clinician, if there are factors that prevent an immediate reassignment (e.g. specialized training, staff shortage).

At first opportunity, the manager must find a way for the clinician to switch with another staff member.

Manager/supervisor (accompanied by security, if required) to meet with the patient to communicate the change and specify the following:

- The change in clinician is not due to their request, but a need to protect staff from violence and to establish a workplace free of discrimination and harassment
- All UHN staff are capable and qualified to deliver excellent care
- The patient is expected to conduct themselves in accordance with the patient bill of rights and responsibilities while at UHN

Manager/supervisor to document request and subsequent discussion in patient chart

Manager to ensure that the clinician is made aware of / provided with various support services (i.e., Spiritual Care, EFAP, IDEAA, WSIB, Bioethics, UHN Wellness)

- Debrief with the unit / larger team, if affected clinician desires
- Debrief with the affected clinician individually, if desired. Offer virtual debrief options.

Inform new clinician about transfer of care and the patient's statements / behaviour

- Manager/supervisor to communicate reason for change with unit staff in a sensitive manner

If a learner was the target of the biased request, preceptors should consider if they are able to supplement the missed learning opportunity elsewhere.

- If affected clinician is a learner, trigger all learner resources and supports within UHN and home institution / school.

Table of Acronyms

Acronym	Meaning
**AOS**	Administrator on Site
**BSA**	Behaviour Safety Alert
**CL Psych**	Consult Liaison Psychiatry
**ECP**	Essential Care Partner
**ED**	Emergency Department
**EFAP**	Employee and Family Assistance Program
**HCCA**	Health Care Consent Act
**IDEAA**	Inclusion, Diversity, Equity, Accessibility and Anti-Racism
**IHP**	Indigenous Health Program
**MRP**	Most Responsible Physician
**PG&T**	Office of the Public Guardian and Trustee
**SDM**	Substitute Decision Maker
**WSIB**	Workplace Safety and Insurance Board

UHN Ethics

For further information or inquiries on how to use this algorithm, please contact the Department of Clinical and Organizational Ethics.

Open in UHN Corporate Intranet
Department of Clinical and Organizational Ethics
