# Supplementary material for: Navigating discriminatory requests and refusals of healthcare workers: A Canadian-based inpatient hospital algorithm
Source: Nurs Ethics. 2025 Sep 6;33(2):344–64. doi: 10.1177/09697330251374153 (PMC13009228; doi:10.1177/09697330251374153)
Supplement: Supplemental Material—Navigating discriminatory requests and refusals of healthcare workers: A Canadian-based inpatient hospital algorithm [file sj-pdf-2-nej-10.1177_09697330251374153.pdf]

Appendix B

Sample responses to patients/their family members/ECPs/visitors regarding hateful language or biased behaviour

Response to the patient:

- "[Patient's name], that kind of language and/or behaviour is not accepted here. When you use that language and/or do [behaviour] it is offensive/unkind to me and those around us. While at UHN, you have access to great healthcare workers who are here to help you and who are all well equipped to do so. We want to provide you with excellent care, but we need to be safe in order to do that. When you use this kind of language or behave in this way, it makes us feel unsafe in this environment and makes it hard for us to do our job. I will remind you that the Patient Bill of Rights and Responsibilities states that discriminatory language and behaviour towards healthcare workers is not tolerated, and it is expected that you abide by this while at UHN. We will continue to do our best to take care of you and in return, ask that you treat every member of our team with the respect and dignity they deserve."

Response to family members/visitors:

- "[Individual's name], we want to provide great care for [name of patient], however, when you use this kind of language/behave in this way, it makes us feel unsafe and makes it hard for us to do our job. That kind of language and/or behaviour is offensive/unkind. Discriminatory language or behaviour towards healthcare workers is not tolerated. While you are here, we expect you to treat every member of our team with the respect and dignity they deserve. If this behaviour persists or continues, we may need to consider changes to your visitation abilities in order to protect our team from continued exposure such incidents."

Probing Questions for Managers to Ask Patients/Family/Visitors who Request a Specific Clinician or Refuse Care from a Specific Clinician

1. I understand you've requested a change in clinician, could you tell me more about why you'd like this change?
2. You've indicated you want a change (specifically X), could you share the reasons for this request?
3. Do you see this change as having an effect on your care journey?
4. Why do you see this change as necessary for the care that you receive here?
5. Are there any other things we need to consider with respect to your request for accommodation?

Sample Prompts for HCW's Unpacking a Patient's Biased Statement/Comment

1. "I heard you say ___. Can you explain what you meant by that?"
2. "What concerns you about _________?"
3. "Can you help me understand what you mean by that statement/comment."

Sample Prompts for HCW's Responding to Patient's Biased/Discriminatory Comments in the Moment

1. "At UHN, I am/we are here to focus on your health. This type of language and/or behaviour goes against the values that we uphold here. We are going to come back in 30 minutes and hope you will be ready to focus on your health"
2. "At UHN, your care team is made up of many different people, all working to improve your health. We treat people with dignity and respect here, and I ask you to do the same."
3. "This clinic is an area where we treat one another with respect. We do not tolerate any discrimination."
4. "That kind of language isn't very nice. I believe a more inclusive term is ___."
5. "That stereotype isn't fair. X is a capable and intelligent provider who is working hard to care for you."

6. “All UHN team members are very qualified. Our top priority is that you receive the best care.”
7. “We are confident in X’s clinical skills.”
8. “UHN wants to provide you with excellent care and X is the right person to do so.”
9. “You are partnered with an amazing provider and are so fortunate to have them taking care of you.”
10. “I must remind you that our Patient Bill of Rights and Responsibilities outlines that discriminatory language and behaviour towards staff is not tolerated.”
11. “We are doing our best to take excellent care of you. Please refrain from making racist/sexist/discriminatory statements.”
12. “Your remarks are making it difficult for me to do my job. Please remember that such remarks are not tolerated at UHN.”

Sample Prompts for Managers/Supervisors Leading Debriefs with Affected Clinicians/Unit Members

1. “The patient’s racist language and behaviours were not acceptable. I’m sorry that happened. How can I support you?”
2. “I am really sorry that you experienced that behaviour. It is upsetting; how you are feeling?”
3. “I’m sorry you had to endure that interaction. It is unacceptable. How can I support you?”
4. When you are ready, please let me know if you’d like to talk about what happened or how I can best support you.”
5. “I want to acknowledge how difficult and challenging that interaction was and make room for us to unpack where it has left us/how it has left us feeling.”
6. “I would like to take some time to acknowledge and reflect on how that experience went for everyone.”
7. “I’m really sorry that this happened. What can we do to ensure that you don’t go through that again? How could we have addressed that situation differently to get a better outcome?”
8. “I'm hoping you will share a bit about what went well and what did not go well during that encounter. Perhaps we can identify areas for learning on how best the team can support and respond in the future”
9. “Next time something like this happens, I will…”
10. “I would like us all to commit to protect each other and our environment from the harm of discrimination as much as possible. Can we all agree to that?”
